# Blood Lead Changes during Pregnancy and Postpartum with Calcium Supplementation

**DOI:** 10.1289/ehp.6548

**Published:** 2004-07-27

**Authors:** Brian L. Gulson, Karen J. Mizon, Jacqueline M. Palmer, Michael J. Korsch, Alan J. Taylor, Kathryn R. Mahaffey

**Affiliations:** ^1^Graduate School of the Environment, Macquarie University, Sydney, New South Wales, Australia; ^2^Commonwealth Scientific and Industrial Research Organisation/Division of Exploration and Mining, North Ryde, New South Wales, Australia; ^3^Department of Psychology, Macquarie University, Sydney, New South Wales, Australia; ^4^U.S. Environmental Protection Agency, Office of Prevention, Pesticides and Toxic Substances, Washington, DC, USA

**Keywords:** blood, bone remodeling, calcium supplementation, lead, lead isotopes, postpartum, pregnancy

## Abstract

Pregnancy and lactation are times of physiologic stress during which bone turnover is accelerated. Previous studies have demonstrated that there is increased mobilization of lead from the maternal skeleton at this time and that calcium supplementation may have a protective effect. Ten immigrants to Australia were provided with either calcium carbonate or a complex calcium supplement (~ 1 g/day) during pregnancy and for 6 months postpartum. Two immigrant subjects who did not conceive acted as controls. Sampling involved monthly venous blood samples throughout pregnancy and every 2 months postpartum, and quarterly environmental samples and 6-day duplicate diets. The geometric mean blood lead at the time of first sampling was 2.4 μg/dL (range, 1.4–6.5). Increases in blood lead during the third trimester, corrected for hematocrit, compared with the minimum value observed, varied from 10 to 50%, with a geometric mean of 25%. The increases generally occurred at 6–8 months gestation, in contrast with that found for a previous cohort, characterized by very low calcium intakes, where the increases occurred at 3–6 months. Large increases in blood lead concentration were found during the postpartum period compared with those during pregnancy; blood lead concentrations increased by between 30 and 95% (geometric mean 65%; *n* = 8) from the minimum value observed during late pregnancy. From late pregnancy through postpartum, there were significant increases in the lead isotopic ratios from the minimum value observed during late pregnancy for 3 of 8 subjects (*p* < 0.01). The observed changes are considered to reflect increases in mobilization of lead from the skeleton despite calcium supplementation. The identical isotopic ratios in maternal and cord blood provide further confirmation of placental transfer of lead. The extra flux released from bone during late pregnancy and postpartum varies from 50 to 380 μg lead (geometric mean, 145 μg lead) compared with 330 μg lead in the previous cohort. For subjects replete in calcium, the delay in increase in blood lead and halving of the extra flux released from bone during late pregnancy and postpartum may provide less lead exposure to the developing fetus and newly born infant. Nevertheless, as shown in several other studies on calcium relationships with bone turnover, calcium supplementation appears to provide limited benefit for lead toxicity during lactation.

The populations most sensitive to lead exposure from various sources are pregnant women and young children (National Research Council 1993). Ongoing lead exposure not only directly affects health, but also allows the accumulation of lead in tissues such as bone. During pregnancy, the mobilization of bone lead increases, apparently as the bone is resorbed to produce the fetal skeleton (Franklin et al. 1997; Gulson et al. 1997a, 1998b; Manton 1985). Previous studies using only lead concentrations for a limited number of samples (usually three to five) generally showed an increase in blood lead levels during the third trimester, which was attributed to increased bone resorption to meet the calcium requirements of the developing fetus (Hertz-Picciotto et al. 2000; Lagerkvist et al. 1996; Rothenberg et al. 1994; Schell et al. 2000, 2003; Schumacher et al. 1996; Sowers et al. 2002; West et al. 1994).

Recent studies, especially those employing precision stable lead isotope methods, have confirmed the early work of Manton (1985) and demonstrated that extra lead is released from the maternal skeleton during pregnancy and lactation in cynomolgus monkeys (Franklin et al. 1997; O’Flaherty et al. 1998) and in humans (Gulson et al. 1997a, 1998b, 2003; Manton et al. 2003). Furthermore, these studies showed that the lead is transferred to the infant.

Because recent evidence suggests that there are detrimental neurologic effects on young children whose blood lead levels are < 10 μg/dL (Canfield et al. 2003; Lanphear et al. 2000), it is critical to minimize the exposure of the developing fetus and the newborn infant, especially lead from bone.

Results of several cross-sectional studies have indicated that increased levels of calcium during pregnancy have a protective effect, reducing the amount of lead released from bones (Farias et al. 1996; Hernandez-Avila et al. 1996). Calcium carbonate supplementation of 1,200 mg elemental calcium/day gave a modest reduction of 16% in blood lead levels among lactating women with relatively high bone lead burden (Hernandez-Avila et al. 2003). In our longitudinal study of lead mobilization from bone during pregnancy and for 6 months postpartum, we found that both newly arrived migrants to Australia and multigenerational Australian subjects consumed very low amounts of calcium in their diets. Estimations were based on quarterly 6-day duplicate diets and averaged about 400–600 mg calcium/day (Gulson et al. 2001), less than half the intakes recommended by the National Institutes of Health (1994).

Two of the migrant subjects who consumed calcium supplements showed lower amounts of lead mobilized from their skeleton compared with the others on low calcium intakes. In light of this finding and those of the previous studies mentioned above, we undertook a longitudinal investigation in which subjects were provided with calcium supplements during pregnancy and for 6 months postpartum. Our hypothesis was that if calcium supplementation is effective in reducing the mobilization of lead from the maternal skeleton, then there should be little or no change in blood lead or isotopic ratios during pregnancy and postpartum.

## Materials and Methods

We employed the protocols refined during previous investigations (Gulson et al. 1995, 1997a, 1998b).

### Subjects.

Participants were female immigrants to Australia who were of child-bearing age (19–32 years) and whose skeletal lead isotopic composition, based on our previous investigations and an initial blood sample, was different from that in their current environment. In essence, the lead isotopic composition or “fingerprint” in multigenerational Australian residents is different from that in subjects from most other countries, although these differences are reducing over the past decade because of globalization (Gulson 2000; Gulson et al. 2001). Hence, by monitoring migrant subjects who conceive after arrival in Australia, it is possible to detect changes in isotopic composition and lead during pregnancy and the postpartum period arising from increased mobilization of skeletal lead. The approach of using subjects whose skeletal lead was different from that in their current environment to determine exchange phenomenon was first explored by Manton (1977, 1985) and later reiterated by Rabinowitz (1991).

Subjects were recruited by networking through our previous cohort study (Gulston et al. 1998b), from English language classes, and via limited advertising in ethnic newspapers. Compared with the previous cohort, there are some differences in demographics of the subjects in our present study. For example, in the previous cohort, most subjects were from Eastern Bloc countries, such as the former Soviet Union and former Yugoslavia. With changing migration patterns to Australia, subjects in the present study were from South Asia and the Middle East (Table 1). One subject (subject no. 1226) was approximately 4 months pregnant when recruited but was retained because our previous investigations of people from other countries showed that, in all cases, their isotopic compositions were different from that of long-term Australians. Subject 1208 miscarried during the first trimester of her first pregnancy. Subject 1231 returned to China immediately after the birth of her child, so no postpartum results are available. Two nonpregnant control subjects were matched mainly on country of origin as other parameters such as age, parity, and blood lead concentration were found not to be critical in previous studies (Gulson et al. 1997a, 1998b).

Signed consent forms were obtained from each volunteer. This consent form had been reviewed and approved by the ethics committees of the Central Sydney Area Health Service, Western Sydney Area Health Service, and Macquarie University. As part of the entry requirements into Australia, all subjects were declared medically fit.

### Sampling and analysis.

Blood and urine samples were obtained monthly during pregnancy and the postpregnancy period for ≥6 months. The urine samples were collected to back up the blood samples in case isotopic data could not be obtained from the blood samples. Environmental samples of 6-day duplicate diet, drinking water, house dust, gasoline, and ambient air were collected quarterly. High-precision lead isotope ratios and lead concentrations were obtained by thermal ionization mass spectrometry. Details of analytical methods have been published previously (Gulson et al. 1995, 1997a, 1998b).

As in our previous reports (Gulson et al. 1995, 1997a, 1998b), our data are expressed as ^206^Pb:^204^Pb ratios for ease of identifying and illustrating changes, especially for readers with limited experience in isotopic methods and for whom significant differences in the third decimal place of a ratio of, say 0.900, may seem unrealistic. Although Manton and colleagues (2003) express results as ^206^Pb:^207^Pb, direct comparisons of variations can be made between our data because ^206^Pb is the numerator in both ratios. For the statistical analyses, both the measured ^207^Pb:^206^Pb and ^206^Pb:^204^Pb ratios were treated.

Hematocrit was measured in blood samples and creatinine in the urine samples using standard methods.

### Calcium supplements.

Because it was not viable to produce a custom supplement for this study, commercially available Australian calcium supplements, which are typically made from overseas components, were administered to the subjects. Details of the supplements are given in Table 2. The supplements were those with the lowest concentrations of lead and were evaluated as suitable in the following investigation.

Approximately 6 months into the present study, we investigated the impact on blood lead of the calcium supplements to be provided to the subjects. This was necessary to satisfy ourselves that we were not “poisoning” the subjects and their developing fetuses because there had been much recent publicity about the potential toxicity of supplements (Boulos and Smolinski 1988; Bourgoin et al. 1993; Capar and Gould 1979; Rogan et al. 1999; Scelfo and Flegal 2000). This publicity occurred despite earlier work from the 1980s indicating that calcium (plus phosphorus) inhibited the uptake of lead from the gastrointestinal tract. Furthermore, Rogan et al. (1999) discovered that vitamin supplements used in the multi-center lead trial were contaminated with lead. Using a cohort of generally younger adults, we showed that an extra intake of 900–1,000 mg calcium/day for 6 months had minimal impact on the blood lead concentration (Gulson et al. 2001). However, we observed changes in the isotopic composition of the blood with a calcium carbonate supplement indicating that lead from the supplement was entering blood, but at this stage we have no explanation as to why there were no changes in blood lead concentration.

Oral and written instructions regarding dosage were given to the subjects. Compliance relied largely on the loyalty established between the cohort coordinator and the subjects to accurately report compliance, and this was checked monthly by tablet count.

A control group taking a placebo or not taking calcium supplements was decided against because, first, although investigated, producing a placebo for such a small group of subjects was not considered viable; second, the much larger cohort in our previous pregnancy investigations had very low calcium intakes, and the present study was one outcome of this; and third, financial constraints given the expense of running such a project.

### Data treatment.

After a visual inspection of graphic presentations, we analyzed changes in ^206^Pb:^204^Pb (and ^207^Pb:^206^Pb) ratios and blood lead concentration over time from the minimum value of blood lead concentration before an increase during late pregnancy using a regression analysis for time series that took into account any autocorrelation of the residuals from the sequences of observations (the autoregression procedure in SPSS 11.0; Chicago, IL, USA). The autocorrelation was taken into account when calculating the significance and magnitude of time effects (Ostrom 1990).

To assess changes in ^206^Pb:^204^Pb (and ^207^Pb:^206^Pb) ratios and blood lead concentration between pregnancy and postpartum for individual subjects, we used an interrupted time-series analysis procedure (Crosbie 1995). In this analysis, the intercept and slope of the pregnancy (baseline) regression line are jointly compared with those of the regression fitted to the postpartum (treatment) phase, and if the joint test is significant, differences between the two intercepts and two slopes can be considered. This procedure takes account of autocorrelation when fitting the lines and testing the differences, and provides better control of type I error (i.e., an erroneous inference of a significant difference between phases/stages in experiments) than does visual inspection and has acceptable power. It requires measurements at regular intervals, as exemplified by the monthly blood sampling in our investigation.

We calculated the extra amount of lead added to blood during pregnancy and postpartum using a similar area under the curve method that was used for our previous cohort (Gulson et al. 1999). This comparative graphic technique is commonly used for cumulative estimates of lead exposure by integrating blood lead concentration across exposure times in occupational exposure (Chia et al. 1996) or for comparative lead dosings of test animals in the study of such exposure parameters as the relative bioavailability of lead (Casteel et al. 1997). Estimates of extra lead flux mobilized were possible only for migrant subjects whose blood lead concentrations exhibited positive variations during the monitoring period (*n* = 10). We calculated the extra flux by estimating the total area generated by serial blood lead concentrations versus pregnancy and postpregnancy time and subtracting the estimated background area, the portion of the area representing blood lead over time that would have occurred in the absence of added lead releases. Background area was generated from the lowest blood lead level during late pregnancy, in contrast to our previous study (Gulson et al. 1998b), in which the minimum blood lead level usually, although not always, occurred in the first trimester.

## Results

### Changes in blood lead during pregnancy and 6 months postpartum.

**Figure 1:**
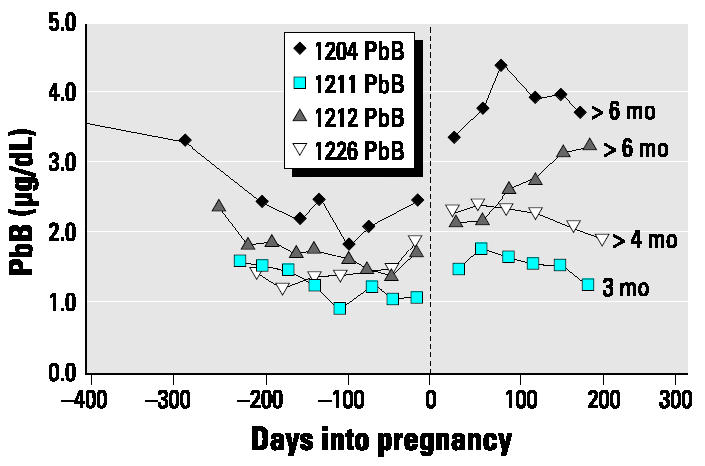
Changes in blood lead concentration (not corrected for hematocrit) for subjects who breast-fed for ≥3 months. The length of breast-feeding is noted to the right of the individual lines. Increases in blood lead concentration occurred in late pregnancy compared with our previous cohort (Gulson et al. 1998b), whose calcium intakes were very low. There are significant increases during the late pregnancy–postpartum period.

**Figure 2:**
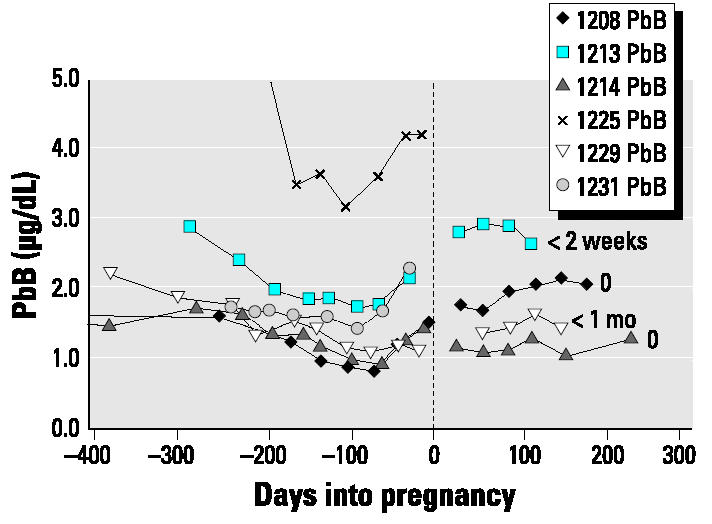
Changes in blood lead concentration (not corrected for hematocrit) for subjects who breast-fed for ≤1 month. The length of breast-feeding is noted to the right of the individual lines. Increases in blood lead concentration occurred in late pregnancy compared with our previous cohort (Gulson et al. 1998b), whose calcium intakes were very low. There are significant increases during the late pregnancy–postpartum period.

The data for the present case series have been subdivided into two groups depending on the length of breast-feeding: those who breast-fed for longer periods (Figure 1), in this case ≥3 months, and those who breast-fed for shorter periods, either not at all or for < 1 month (Figure 2).

**Figure 3:**
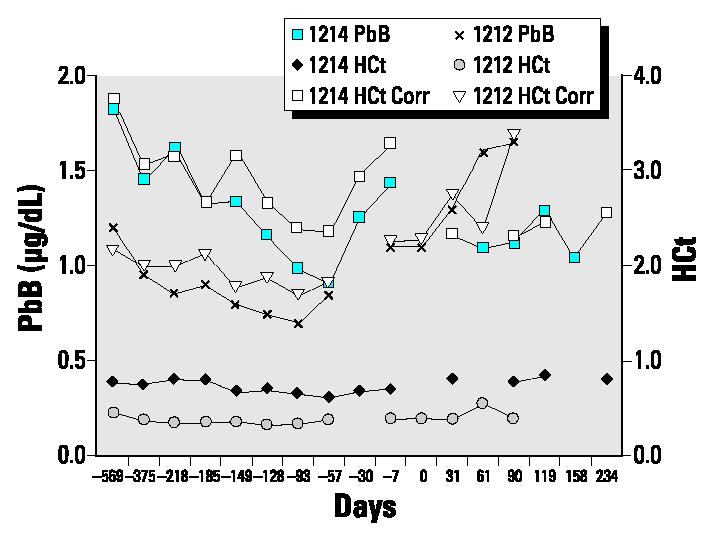
Plot illustrating the changes in hematocrit values (HCt) and uncorrected (PbB) and hematocrit-corrected (HCt Corr) blood lead concentrations for subjects 1212 and 1214. The U-shaped pattern for blood lead concentrations over time persists in the hematocrit-corrected values. The “0 days” scale is offset for subject 1212 and is denoted for all subjects by the breaks in the lines.

To account for the well-recognized changes in blood volume during pregnancy (Hytten 1985), increases of which may result in decreases in blood lead concentration, the blood leads can be adjusted for changes in hematocrit. Data for subjects 1212 and 1214 are shown in Figure 3, which illustrate the changes in hematocrit and the hematocrit-corrected and uncorrected blood lead concentrations over time. Hematocrit values throughout pregnancy in the present case series show decreases, little change, or decreases and then increases in late pregnancy (0–2 months before parturition). As shown in Figure 3, the uncorrected and corrected blood lead values follow similar trends and, because of the “return-to-normal” hematocrit values during the postpartum period and the importance of this period for our results, measured blood lead values are shown in the accompanying figures.

Changes in blood lead during pregnancy followed similar U-shaped patterns for the two groups. From early to mid-pregnancy the hematocrit-corrected blood lead concentrations decreased for 8 of the 10 subjects and for the second pregnancy of subject 1208. During late pregnancy, the blood lead concentrations increased in all subjects (Table 3); the increase in blood lead concentration above a minimum value, corrected for hematocrit, ranged from 10 to 55% (geometric mean, 25%), although the values did not exceed those determined in prepregnancy or early pregnancy.

Changes in blood lead from late pregnancy through the 6 months postpartum were variable and did not appear to be related to length of breast-feeding and calcium intakes. The maximum value during postpartum the concentration was almost double the minimum blood lead value during late pregnancy in half the subjects; the percentage increase varied from 30 to 95%. For half the subjects (subjects 1204, 1211, 1213, and 1226), the blood lead increased until the second or third month postpartum and then either leveled or decreased (Figures 1 and 2). Only in subjects 1212 and 1208 was there a continuous linear increase from late pregnancy through postpartum. The increases postpartum in both groups commonly exceeded the values measured in prepregnancy or early pregnancy.

Autoregression analyses of blood lead concentration from the time of minimum value in late pregnancy through postpartum for eight subjects are presented in Table 4. There are significant positive increases for half the subjects and none for the other four (subjects 1211, 1213, 1214, 1226). However, visual inspection of the plots (Figures 1 and 2) shows that for the latter four, there are increases from late pregnancy to the first 1 or 2 months postpartum and, thereafter, either a leveling or decrease in blood lead.

For the interrupted time-series analyses, the overall test of change was significant for five of eight subjects in blood lead concentration during the whole of the pregnancy phase compared with the postpartum phase (Table 5). There were significant differences in the blood lead slopes for half of the eight subjects during pregnancy compared with postpartum, and for six of eight subjects there were significant differences in intercept.

**Figure 4:**
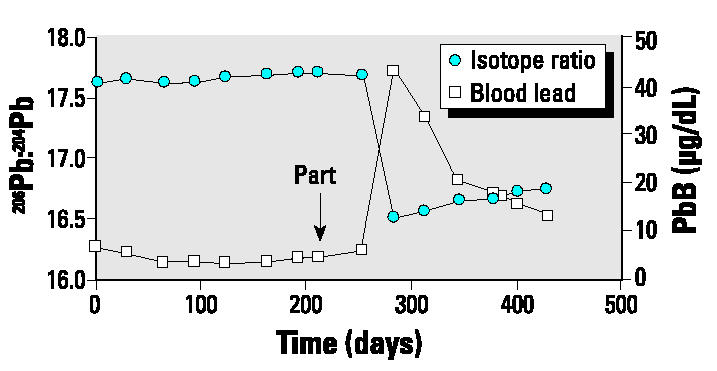
Plot of blood lead concentration (PbB) and isotopic ratios for subject 1225, who experienced a major unknown lead exposure soon after parturition (Part).

Subject 1225 showed a major increase in blood lead concentration soon after parturition, with the value increasing from 5.6 to about 43 μg/dL. This increase was accompanied by a large decrease in the ^206^Pb:^204^Pb ratio toward Australian environmental values (Figure 4).

**Figure 5:**
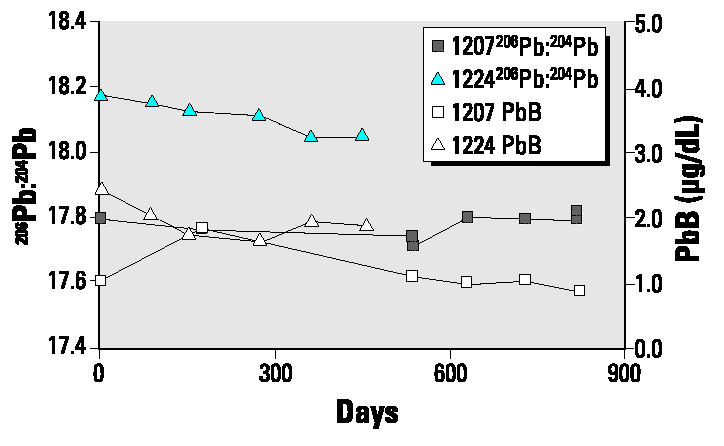
Plot of blood lead concentration (PbB) and isotopic ratios for subjects 1207 and 1224, who acted as nonpregnant controls.

The blood lead concentrations for the controls showed only small variations and an overall decrease over time (Figure 5).

As is well documented in the literature, the cord blood lead concentrations were lower than those of the last maternal sample taken before parturition. The geometric mean ratio of cord:maternal blood lead was 0.72, with a range from 0.55 to 0.93, and the correlation of cord versus maternal blood lead had an *r*^2^ of 0.93.

### Changes in isotopic ratios during pregnancy and 6 months postpartum.

**Figure 6:**
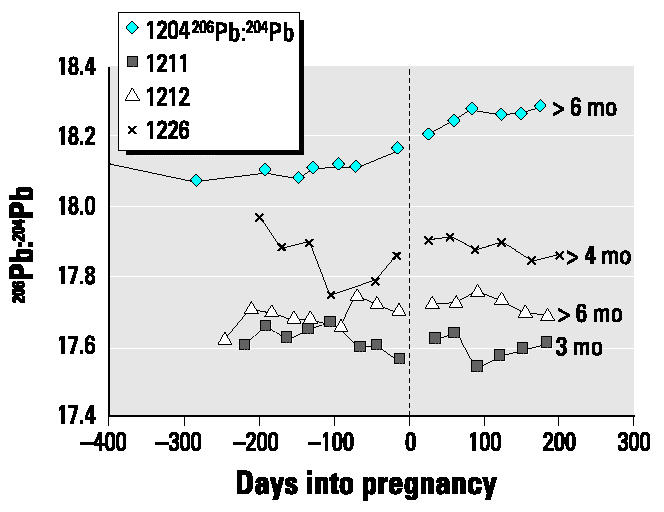
Changes in isotopic ratio expressed as ^206^Pb:^204^Pb for subjects who breast-fed for ≥3 months. The length of breast-feeding is noted to the right of the individual lines.

**Figure 7:**
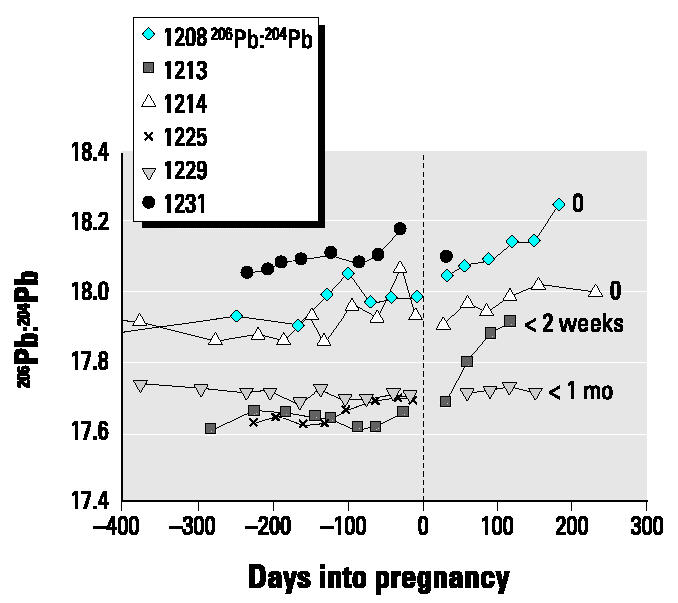
Changes in isotopic ratio expressed as ^206^Pb:^204^Pb for subjects who breast-fed for ≤1 month. The length of breast-feeding is noted to the right of the individual lines.

As was the case for blood lead concentrations, changes in isotopic ratios varied but not with consistent trends (Figures 6 and 7). In some subjects (1212, 1214, 1225, 1229) it was not possible to estimate any changes in isotopic composition during pregnancy, especially late pregnancy, because of a change in the slope or scatter of the data (Table 3). In the other subjects, any changes during late pregnancy ranged from little difference from the experimental error of ± 0.2% up to 0.8% for subject 1226.

Autoregression analyses for the ^206^Pb:^204^Pb (and ^207^Pb:^206^Pb) ratio from the time of minimum value in late pregnancy through postpartum for eight subjects are presented in Table 4. The analyses for both ratios give consistent results, except for subject 1229, although those for the ^207^Pb:^206^Pb ratio tend to show better correlation than those for the ^206^Pb:^204^Pb ratio, probably because of the higher precision and ease of measurement of the ^207^Pb:^206^Pb ratio (~ 0.9) compared with the larger ^206^Pb:^204^Pb ratio. Four of the eight subjects who were monitored during the 6 postpartum months (1204, 1208, 1213, 1229) showed significant increases in the ^206^Pb:^204^Pb ratio, whereas there were minimal changes for the other subjects.

For the interrupted time-series analyses, in no case was there a significant overall test of change for ^206^Pb:^204^Pb (and ^207^Pb:^206^Pb ratio; not shown in Table 5), nor was there any significant difference in the slopes or intercepts for the isotopic ratios (Table 5).

There were small variations in the isotopic compositions of the control subjects (Figure 5). The ratios for the Bulgarian subject decreased slowly, reflecting an ongoing exchange between her long-term body stores and Australian environmental lead, as we have observed in migrant subjects who did not conceive (Gulson et al. 1997b).

Isotopic ratios in the cord blood samples were similar to those in the maternal blood samples taken before parturition (*r*^2^ = 0.98), as we found in our previous studies (Gulson et al. 1998b) and also observed by Manton (1977, 1985). The similarity in isotopic ratios of the maternal and cord blood samples provides confirmation of the placental transfer of lead.

### Dietary samples.

Lead concentrations in duplicate diet samples vary considerably both between and within subjects. For example, the geometric mean concentration for all subjects was 7.1 μg/kg with a range of 3.0–20.1 μg/kg (*n* = 48). In contrast, the range in ^206^Pb:^204^Pb ratio was surprisingly less than expected, from 17.79 to 18.81, with a geometric mean ratio of 18.25.

There does not appear to be any relationship between calcium intake and changes in isotopic composition of blood lead during pregnancy and postpartum. For example, subjects 1208 and 1213 exhibited the largest increases in ^206^Pb:^204^Pb ratio and had a high compliance with calcium supplements. Likewise, subjects 1204 and 1212 had a high compliance and showed large increases in blood lead concentration postpartum. There was also no relationship between the time of increase in blood lead during pregnancy and calcium intake.

### Environmental samples.

Dustfall accumulation ranged from 6 to 94 μg/m^2^/month with a geometric mean of 17 μg/m^2^/month (*n* = 36). The ^206^Pb:^204^Pb ratios ranged from 16.50 to 17.85 and were similar to values measured in Sydney air from high-volume air filter samples (Chiaradia et al. 1997); the geometric mean was 17.16.

Lead concentration in fully flushed drinking water ranged from 0.03 to 1.1 μg/dL with a geometric mean ^206^Pb:^204^Pb ratio of 16.80; at these very low concentrations, water has no impact on blood lead.

## Discussion

### Changes in blood lead concentration and isotopic ratios during pregnancy and 6 months postpartum.

Mean increases of approximately 25% in blood lead concentration during late pregnancy for our present case series with calcium supplementation are consistent with increases observed in our previous cohort (Gulson et al. 1998b) with low calcium intakes and in several other studies (Hertz-Picciotto et al. 2000; Lagerkvist et al. 1996; Rothenberg et al. 1994; Schell et al. 2000, 2003; Schumacher et al. 1996; Sowers et al. 2002; West et al. 1994). However, the increases found in the present study occurred later in pregnancy than in our previous cohort (Gulson et al. 1998b) and in many of the other studies (Hertz-Picciotto et al. 2000; Lagerkvist et al. 1996; Rothenberg et al. 1994; Schell et al. 2000, 2003; Schumacher et al. 1996; Sowers et al. 2002; West et al. 1994).

The changes in blood lead during the whole of pregnancy follow a U-shaped curve, as previously observed by Rothenberg et al. (1994), Hertz-Picciotto et al. (2000), Sowers et al. (2002), and Schell et al. (2000, 2003). Apart from the recent study of Manton et al. (2003) and our investigations (Gulson et al. 1995, 1997a, 1998b), only limited numbers of samples were collected in the other studies, usually a maximum of one for each trimester, one at delivery, and one postpartum. In our present case series, the changes in blood lead during pregnancy were obvious whether the blood lead values were corrected or uncorrected for hematocrit. Hematocrit-corrected values did not alter the findings of Hertz-Picciotto et al. (2000) and Schell et al. (2000), but in the case of Schumacher et al. (1996), the decrease in uncorrected blood lead in the first trimester compared with prepregnancy was not observed for the hematocrit-corrected values. Furthermore, blood lead values in the second trimester (~ 20 weeks) showed average increases of approximately 14%, and for the maximum increase observed in the 32-week sampling, the change from the minimum value at 8 weeks was 36%. Sowers et al. (2002) observed increases in blood lead from the 20th week to delivery in African Americans (1.20–1.49 μg/dL; an increase of 24%) and Hispanic Americans (0.99–1.32 μg/dL; an increase of 33%) but a barely detectable decrease in blood lead of 0.03 μg/dL at delivery for Caucasians. Schell et al. (2003) observed smaller increases in blood lead from the second trimester to delivery in whites (1.6–1.8 μg/dL; an increase of 12%) compared with African Americans (1.8–2.5 μg/dL; an increase of 39%).

Four of the eight subjects in the present case series showed linear increases in blood lead concentration from the minimum in late pregnancy through postpartum, but there was no consistency with length of breast-feeding or compliance with calcium supplementation. Manton et al. (2003) also found major increases in blood lead concentration by up to a factor of 3 from a minimum value during pregnancy to postpartum. The magnitude of the increases was attributed partly to breast-or bottle-feeding by the mothers. In contrast to the changes observed in the above investigations, Berglund et al. (2000) could not detect any increase in blood lead during pregnancy, but they found a significant increase in blood lead concentration during lactation.

### Extra blood lead and calcium supplementation.

For our present case series, with subjects taking calcium supplements, the increases in blood lead from the minimum value during late pregnancy took place at about 6–8 months in all subjects. In contrast, for the previous cohort (Gulson et al. 1998b), whose daily calcium intake was generally very low, an increase in blood lead from the minimum occurred at 3–4 months for six of the nine subjects. Thus, even though there is an increase of similar magnitude in blood lead from a minimum value during pregnancy in both these cohorts, the fetus in the group receiving calcium supplements would be exposed to considerably lower lead flux than would those whose mothers had a low calcium intake. This is confirmed by the lower mean value of 145 μg lead for the extra flux to blood during late pregnancy through postpartum for the present higher-calcium-intake subjects compared with the mean of 330 μg lead for the low-calcium-intake cohort. In breast-feeding mothers who were consuming close to, or exceeding, what they recommended as the daily calcium intake of 1,000 mg/day, Manton et al. (2003) suggested there was no tendency for increases in blood lead in late pregnancy. However, inspection of their Figure 2 shows that there are increases in blood lead concentration in the last 2 months of pregnancy; increases in blood lead in late pregnancy also occurred for at least four bottle-feeding mothers with varying calcium intakes, consistent with our observations.

We argued that the changes in blood lead concentration and isotopic composition during pregnancy and postpartum in our previous cohort (Gulson et al. 1998b) reflected increased mobilization of lead from the maternal skeleton, probably associated with the low calcium intakes of the subjects (Gulson et al. 1998b). Any changes in blood lead concentration during pregnancy and postpartum are a function of plasma volume, red cell mass, and variations in lead flux from endogenous and exogenous sources. Low blood lead concentrations during the first half of pregnancy have been attributed to hemo-dilution (Rothenberg et al. 1994), which has been associated mainly with an increased plasma volume, calculated by Hytten (1985) to be approximately 1,250 mL for a normal pregnancy. Hytten (1985) showed that once the “plateau” of increased plasma volume had been attained at about week 30 of pregnancy, there was minimal increase in plasma volume and, as expected, little change during postpartum. That is, the dilution effects of increased plasma volume on blood lead concentration should be most marked during the second and early third trimesters. Hytten (1985) also drew attention to changes in red cell mass and the impact of iron supplementation on this. The changes in volume are mirrored in hematocrit, which decreases until third trimester and then increases above normal values until early in postpartum (Figure 3; also see Schell et al. 2000, their Figure 2). During pregnancy, if the flux of lead into blood remains constant, blood lead concentration will decrease as blood volume increases. On the other hand, if blood lead concentrations remain constant or increase during late pregnancy, this suggests that there has been an increase in lead flux into the blood.

The decreases in blood lead from early to mid-pregnancy in our present and previous cohort (Gulson et al. 1998b) are consistent with a hypothesis of reduced flux from the skeleton, as indicated by our estimations of flux. Such a reduction in lead flux was noted by Franklin et al. (1997) in cynomolgus monkeys and in one subject in the recent study of Manton et al. (2003). Although they did not report data from early pregnancy, Manton et al. (2003) hypothesized that in early (to mid) pregnancy, only trabecular bone of presumably low lead content was resorbed, decreasing lead concentrations more than expected from hemo-dilution alone. In late pregnancy, more cortical bone with a presumed higher lead content was resorbed, increasing blood lead concentrations. Such a hypothesis would appear to conflict with measurements of bone lead over the past decades showing that patella or calcaneus (trabecular) bone has higher concentrations than does tibia (cortical) bone (e.g., Hernandez-Avila et al. 2002; Oliveira et al. 2002; Rothenberg et al. 2000) and that the trabecular/cortical bone lead ratio ranges from 1 to 2. Nevertheless, the total body burden of lead in cortical bone is generally higher than in trabecular bone because the proportion of cortical:trabecular bone is about 4:1.

### Bone research of relevance to the changes in lead during pregnancy and lactation.

The linear increases in blood lead isotopic ratio, blood lead concentration, and increased lead flux to blood during late pregnancy and postpartum in half the subjects from the present case series and most subjects in our previous cohort (Gulson et al. 1998b) are attributed to increased flux from the maternal skeleton, despite increased calcium intake for the present case series.

Rather than cortical bone providing the major contribution to increased blood lead, as suggested by Manton et al. (2003), it may well be trabecular bone. For example, based on bone X-ray fluorescence measurements, Schütz et al. (1987) and Hu et al. (1989) suggested that trabecular bone exerted the most influence on blood lead and that this was consistent with the higher vascular and turnover rates associated with trabecular compared with cortical bone.

Bone mineral density and biochemical markers of bone turnover and especially resorption during pregnancy and lactation appear to affect trabecular more than cortical bone (Black et al. 2000; Kolthoff et al. 1998; Naylor et al. 2000; Sowers et al. 2000) as a result of having more cancellous (trabecular) bone surfaces available for turnover (Sowers et al. 2000). Even though Sowers et al. (2000) stated that findings of previous studies were inconsistent, there was a convergence toward bone loss during pregnancy and lactation. For example, there are findings of increase at localized bone sites (Drinkwater and Chestnut 1991), no change (Sowers et al. 1991), and bone loss (Aguado et al. 1998; Honda et al. 1998; Kolthoff et al. 1998; Lamke et al. 1977; Laskey et al. 1998; Naylor et al. 2000; Pires et al. 2002; Ritchie et al. 1998; Sowers et al. 1995). Lactation would appear to be associated with a larger decrease in bone density and net bone loss of from 1 to 5% during 3–6 months postpartum compared with pregnancy (Laskey et al. 1998; Pires et al. 2002; Ritchie et al. 1998; Sowers et al. 1995).

Bone research can provide further useful information with respect to the observation in lead pregnancy studies that the most significant increase in blood lead concentration (and significant changes in isotopic ratios) occurs in late pregnancy. For example, Sowers et al. (2000) confirmed that there was increased bone turnover in the third trimester of pregnancy compared with the first trimester, as determined by measurement of the urinary excretion of markers of type I collagen, but suggested that the mechanisms of bone resorption during pregnancy are poorly understood. Furthermore, the data of Black et al. (2000) and Naylor et al. (2000) indicated that there was a dissociation of bone formation and resorption in the first two trimesters and well into the third trimester of pregnancy. They suggested that the gain in bone mineral density at cortical bone sites during pregnancy may result from the redistribution of mineral from trabecular to cortical bone sites and that elevated bone turnover may explain trabecular bone loss during pregnancy. On the other hand, a study of primates using the stable lead isotope method indicated that there was a reduction in first-trimester bone mobilization (Franklin et al. 1997). These conclusions are, however, inconsistent with data from bone mineral density and bone turnover indices demonstrating that bone resorption increased in the first trimester. For example, the bone biopsy data of Purdie et al. (1988) in women at the time of termination of pregnancy at 12–14 weeks of gestation showed that bone resorption predominated in early pregnancy. Likewise, Shahtaheri et al. (1999) reported early bone loss in bone biopsy samples from 15 women in their first trimesters, whereas at term in another 13 women they found new and more numerous (but thinner) trabeculae.

### Bone turnover and calcium and lead relationships during pregnancy and lactation.

Changes in blood lead during pregnancy and lactation are inexorably linked with changes in calcium. During pregnancy and lactation, there is increased demand for calcium for transport to the fetus. The maternal response to the demand for calcium theoretically can involve increased absorption of calcium from the intestine, greater calcium conservation by the kidneys, or greater bone turnover (Sowers et al. 2000). There are, however, wide disparities in the literature on both bone/calcium and lead about the efficacy of calcium intakes during pregnancy and lactation.

Several authors consider that bone mineral changes especially during lactation are hormonally regulated and independent of the amount of calcium in the woman’s diet; provision of additional calcium has minimal impact on preventing bone resorption at sites such as the spine and femur (Cross et al. 1995; Kalkwarf 1999; Kolthoff et al. 1998; Laskey et al. 1998; Prentice 2000). For example, Prentice (2000) states there are firm data demonstrating that a low calcium intake during lactation does not lead to impaired lactational performance or to exaggerated bone loss.

In contrast, previous evidence suggested that bone loss during lactation in adolescents may be prevented by adequate dietary calcium intakes (Chan et al. 1987). Later on, Krebs et al. (1997) suggested that bone loss during lactation may be attenuated by a generous dietary ratio of calcium to protein. Specker et al. (1994) concluded that even when dietary intake of calcium exceeded the recommended daily intake, the calcium demands in lactation in humans were preferentially met by increased skeletal resorption of calcium and probably increased renal conservation of calcium, but not by increased intestinal absorption of calcium.

Like the complexity in the bone/calcium literature, the relationships between bone lead, calcium, bone turnover, and reproduction are also controversial. For example, Rothenberg et al. (2000) found calcaneus and tibia lead were directly associated with prenatal third trimester blood lead but only calcaneus lead was associated with postnatal blood lead. They also found that there was no effect of dietary calcium on calcaneus lead despite the more easily mobilized trabecular lead. In a cross-sectional Mexico City study, Hernandez-Avila et al. (1996) also found a significant association between trabecular bone lead (in the patella) and postnatal blood lead and a significant effect of calcium on patella lead.

With respect to calcium intakes, several studies have suggested that dietary calcium may have a protective role against lead by decreasing absorption of lead in the gastrointestinal tract and by decreasing the mobilization of lead from bone stores to blood, especially during periods of high metabolic activity of the bone such as pregnancy, lactation, and menopause (e.g., Farias et al. 1996; Hernandez-Avila et al. 1996, 1997, 2003; Hertz-Picciotto et al. 2000). However, most of these studies were cross-sectional in nature, and dietary calcium was estimated by questionnaire, diary, or recall. Furthermore, the outcomes between studies of similar population groups were sometimes conflicting (e.g., Farias et al. 1996; Hernandez-Avila et al. 1996). In more recent studies in Mexico City, calcium carbonate supplementation of 1,200 mg elemental calcium per day gave a modest reduction of 16% in blood lead levels among lactating women with relatively high bone lead burden (Hernandez-Avila et al. 2003). In an earlier report apparently using the same cohort but with lower numbers, there did not seem to be any benefit from calcium supplementation (Tellez-Rojo et al. 2002). Using a sensitive biomarker of bone resorption, levels of cross-linked N-telopeptides of type I collagen (NT_x_), Janakiraman et al. (2003) observed that a bedtime 1,200 mg calcium supplement during the third trimester of pregnancy reduced maternal bone resorption by an average of 14%.

### Contribution to blood lead from diet and calcium supplements.

In contrast to our previous cohort (Gulson et al. 1998b), where the environmental and dietary contributions to blood lead were considered minimal, conditions were somewhat different in the present case series. For example, even though the ^206^Pb:^204^Pb ratios in the dust, air, and water are still lower than observed in the blood of our migrant subjects and would contribute little to blood lead, the ratios in the 6-day duplicate diet increased considerably, especially since our market basket surveys of 1990 when the ^206^Pb:^204^Pb ratio was about 17.0 (Gulson et al. 1996). This change has come about with globalization, especially of the food supply. Nevertheless, we do not attribute the increase in blood lead during the last 2 months of pregnancy to diet; otherwise, there should have been obvious effects earlier in the pregnancy. A potential source of the increased lead may come from the calcium supplements. These contribute almost 50% of the daily lead intake, and even though we observed minimal changes in the 6-month trial of a separate cohort taking the same dose and same type of calcium supplements (Gulson et al. 2001), physiologic processes during pregnancy may affect the uptake of lead even in the presence of sufficient calcium. For example, pregnant swine absorb and retain more lead than do nonpregnant swine (Casteel et al. 2001), and there is convincing evidence especially from stable calcium isotope studies, mentioned above, that there is increased intestinal absorption of calcium during pregnancy. An increased absorption of lead from the calcium supplement could partially explain the increase in ^206^Pb:^204^Pb ratio observed for three subjects during 6 months postpartum, but there was no such change in the other three subjects for whom we have data. Furthermore, there was no consistency in changes relating to the two different supplements.

The major decrease in ^206^Pb:^204^Pb ratio and increase in blood lead for subject 1225 postpartum are remarkable (Figure 4). Fortunately, this subject was not breast-feeding. A sample of the husband’s blood was collected approximately 4 months after detection of the changes in his wife; it had a lead concentration of 23 μg/dL and a ^206^Pb:^204^Pb ratio of 16.62, consistent with an exposure similar to that of the wife. Considerable effort was devoted to determine the source of the changes because they indicate a very high acute dose of lead of Australian origin. However, evaluation of the potential sources (diet, traditional medicines, cosmetics, social calendar) indicated that the only explanation for the changes could be attributed to their attendance at an ethnic fair where they consumed foodstuffs different from their regular diet.

In summary, despite the recent studies of Hernandez-Avila et al. (2003) and Janakiraman et al. (2003) pointing to benefits from calcium supplementation during pregnancy and lactation, significant increases in blood lead during late pregnancy and postpartum found in our investigations and those of several other authors appear to indicate that calcium supplementation is ineffective in minimizing the mobilization of lead from the skeleton during lactation—a position consistent with evidence from the calcium literature using bone density and bone turnover index measurements (Prentice 2003). It does appear to offer some protection, however, during pregnancy by delaying the extra lead mobilized from bone and reducing the extra flux. Despite the potential exposure of the infant to lead from breast-feeding, we have shown that the transfer of lead to the infant from breast milk is low, especially at low blood lead concentrations (Gulson et al. 1998a).

## Figures and Tables

**Table 1:** Subject information.

Subject identifier	Country of origin	Time to conception (months) after arrival	No. of children	Age (years)	Calcium supplement	Average compliance (%) during pregnancy	Initial Pb (μg/dL)	Sex of newborn	Breast-feeding
1204	Bulgaria	13	1	31	Carbonate	68	4.0	M	> 6 months
1207*^a^*	Croatia	NA	1	26	NA	NA	1.0	NA	NA
1208*^b^*	Bosnia	34	1	23	Carbonate	73	1.9	M/F	0
1211*^c^*	Bangladesh	8	1	25	Carbonate	95	1.6	M	3 months
1212*^c^*	Turkey	20	1	31	Carbonate	86 (1st 2 months)	2.4	F	> 6 months
1213	Lebanon	4	0	32	Complex	71 (1st 6 months)	2.9	M	< 2 weeks
1214	Turkey	14	0	25	Complex	52	1.8	F	0
1224*^a^*	Bulgaria	NA	0	31	NA	NA	2.4	NA	NA
1225*^c^*	Pakistan	3	0	28	Complex	72	6.5	F	0
1226^c^,^d^	Iraq	1	0	20	Complex	56	1.4	M	> 4
1229	Lebanon	18	0	19	Complex	52	2.3	M	< 1 month
1231^a^,^e^	China	2	0	32	Carbonate	100 (6 months)	1.7	F	Unknown

NA, not applicable.

a Nonpregnant controls.

b Gave birth to twins.

c Pregnant when recruited.

d About 4 months pregnant on recruitment

e Subject 1231 returned to China immediately after giving birth.

**Table 2:** Product information.

	Product 1 (complex product)			Product 2
Composition	Calcium citrate	Calcium phosphate	Calcium amino acid	Calcium carbonate +vitamin D_3_
Weight Ca compound (mg)	300	325	200	1,500
Equivalent Ca (mg)	64	126	40	600
Daily total Ca (mg)		920		1,200
Daily dosage		3 times after meals, 1 on retiring		Twice daily
^206^Pb:^204^Pb		20.1		18.5
Pb [μg/kg (μg/tablet)]		293 (0.4)		940 (1.6)
Daily Pb intake (μg)		2.8		3.2

**Table 3:** Changes during pregnancy and postpartum.

			Percent increase in late pregnancy		Percent increase postpartum			
Identifier	Country of origin	Month PbB increased > minimum*^a^*	PbB (HCt corr)	^206^Pb:^204^Pb*^b^*	PbB	^206^Pb:^204^Pb*^b^*	Percent increase in PbB:min value*^c^*	Extra flux*^d^* (μg)
1204	Bulgaria	8	40	0.17	30	0.33	90	380
1208	Bosnia	8	55	0.11	40	1.22	95	150
1211	Bangladesh	6	10	0.51	50	ND	90	125
1212	Turkey	8	10	ND	55	ND	95	235
1213	Lebanon	8	25	0.28	30	1.65	70	140
1214	Turkey	8	40	ND	10	0.61	40	80
1225	Pakistan	6	50	ND	NA	NA	NA	NA
1226	Iraq	8	20	0.80	20	ND	50	200
1229	Lebanon	6	10	ND	40	0.2	30	50
1231	China	4	40	0.33	NS	NS	NA	165*^a^*

Abbreviations: (HCt corr), (hematocrit corrected); min, minimum; NA, not applicable; ND, not able to be estimated because of change in slope (see figures); NS, no samples; PbB, blood lead concentration.

a During pregnancy.

b Relative to experimental error of ± 0.2%.

c Late pregnancy to postpartum.

d Extra lead released from bone during late pregnancy and postpartum.

**Table 4:** Results for autoregression analyses.

	*R*^2^-value			*p*-Value		
Subject	^206^Pb:^204^Pb*^a^*	^207^Pb:^206^Pb	PbB	^206^Pb:^204^Pb	^207^Pb:^206^Pb	PbB
1204	0.87 (10)	0.91	0.63 (9)	< 0.001	< 0.001	0.007
1208	0.94 (9)	0.94	0.85 (9)	< 0.0001	< 0.00001	< 0.001
1211	0.12 (11)	0.25	0.22 (10)	0.32	0.15	0.38
1212	0.09 (9)	0.23	0.94 (8)	0.46	0.25	0.002
1213	0.91 (7)	0.85	0.40 (6)	0.006	0.01	0.30
1214	0.12 (10)	0.60	0.09 (9)	0.35	0.07	0.37
1226	0.04 (9)	0.16	0.14 (8)	0.56	0.31	0.45
1229	0.76 (8)	0.28	0.77 (7)	0.002	0.21	0.002

Numbers of data points in analysis are given in parentheses.

a Cord blood value incorporated in analysis, except for subject 1208 (no sample available).

**Table 5:** Results for interrupted time-series analyses.

	No. of measures		Sum squares intercept		Sum squares slope		Overall test of change	
Subject	Pregnancy	PP	*t*-Value	*p*-Value	*t*-Value	*p*-Value	*F*-value	*p*-Value
1204	7	7	1.40	0.194	0.45	0.664	0.14 (2,9)	0.87
	8	6	4.12	0.003	–3.14	0.012	5.75 (2,9)	0.025
1208	7	6	0.52	0.617	1.41	0.197	1.16 (2,8)	0.36
	7	6	4.79	0.001	–3.35	0.010	5.81 (2,8)	0.028
1211	8	7	–1.94	0.081	1.41	0.188	1.28 (2,10)	0.32
	8	6	2.50	0.034	–2.15	0.06	4.91 (2,9)	0.036
1212	9	7	2.03	0.068	–1.45	0.175	2.01 (2,11)	0.181
	9	6	2.70	0.022	–1.21	0.255	0.83 (2,10)	0.464
1213	9	5	–0.11	0.915	1.39	0.197	1.50 (2,9)	0.274
	9	4	4.77	0.001	–4.0	0.004	8.01 (2,8)	0.012
1214	10	7	1.23	0.241	0.22	0.828	3.60 (2,12)	0.060
	10	6	–1.21	0.253	1.04	0.320	0.64 (2,11)	0.547
1226	6	8	1.57	0.15	–1.02	0.34	1.05 (2,9)	0.39
	6	7	3.94	0.004	–4.39	0.002	10.27(2,8)	0.006
1229	8	5	–0.05	0.964	1.11	0.301	1.09 (2,8)	0.380
	8	4	1.32	0.229	–0.76	0.474	1.10 (2,7)	0.385

PP, postpartum. For all subjects, first row is ^206^Pb:^204^Pb and second row is PbB. Values in parentheses are degrees of freedom. Cord blood value incorporated in postpartum analysis, except for subject 1208 (no sample available).
